# Extra-facial Melasma Presenting With Isolated Forearm Involvement in a Male Patient

**DOI:** 10.7759/cureus.108919

**Published:** 2026-05-15

**Authors:** Daniella M Allevato, Thomas Davis, Dathan Hamann

**Affiliations:** 1 Dermatology, HonorHealth, Scottsdale, USA; 2 Dermatopathology, Sagis Diagnostics, Houston, USA; 3 Dermatology, Saguaro Dermatology, Phoenix, USA

**Keywords:** extra-facial melasma, forearm hyperpigmentation, male melasma, occupational sun exposure, photodamage, pigmentary disorders, skin of color, ultraviolet radiation

## Abstract

Melasma is an acquired disorder of hypermelanosis that most commonly affects sun-exposed facial skin in women. Extra-facial involvement and occurrence in male patients are uncommon. We present a case of a 66-year-old Hispanic male with asymptomatic, symmetric hyperpigmented patches on the bilateral forearms in the setting of chronic occupational ultraviolet (UV) exposure as a truck driver. Notably, the patient had a history of severe facial burns two years prior to presentation, with complete sparing of facial skin hyperpigmentation. A punch biopsy demonstrated increased basal layer melanin pigmentation with few dermal melanophages and no significant inflammatory infiltrate, consistent with epidermal melasma. The patient was treated with topical azelaic acid and photoprotection counseling but reported minimal improvement, likely related to ongoing UV exposure and occupational constraints. This case highlights an uncommon presentation of extra-facial melasma in a male patient and suggests that prior cutaneous injury may influence melanocytic responsiveness. It also emphasizes the role of chronic UV exposure in disease distribution and persistence.

## Introduction

Melasma is an acquired disorder of hypermelanosis characterized by localized, symmetric hyperpigmented macules and patches with irregular, moth-eaten borders. It most commonly affects sun-exposed areas, particularly the face, including the malar, centrofacial, and mandibular regions. The condition is more prevalent in individuals with darker skin phototypes, especially Fitzpatrick skin types III-V, and is frequently observed among Hispanic, Asian, and African American populations [1].

Epidemiologic studies demonstrate a strong female predominance, with women comprising up to 97.5% of cases. Additionally, extra-facial melasma has been reported more commonly in postmenopausal women, with a prevalence of approximately 14.2% in this subgroup [2]. In contrast, melasma in men is uncommon, and one study conducted in Puerto Rico reported men accounting for approximately 10% of cases in reported series [3].

Although the exact pathogenesis of melasma remains incompletely understood, multiple factors contribute, including ultraviolet (UV) radiation, hormonal influences (such as pregnancy, oral contraceptive use, and hormone replacement therapy), thyroid dysfunction, inflammatory bowel disease, use of cosmetics, photosensitizing medications, genetic susceptibility, and environmental exposures. UV radiation is a central driver of melanogenesis [4], while estrogen-mediated upregulation of tyrosinase activity also contributes to pigment production [5]. Occupational UV exposure is increasingly recognized as a significant exacerbating factor in susceptible individuals [6].

## Case presentation

**Figure 1:**
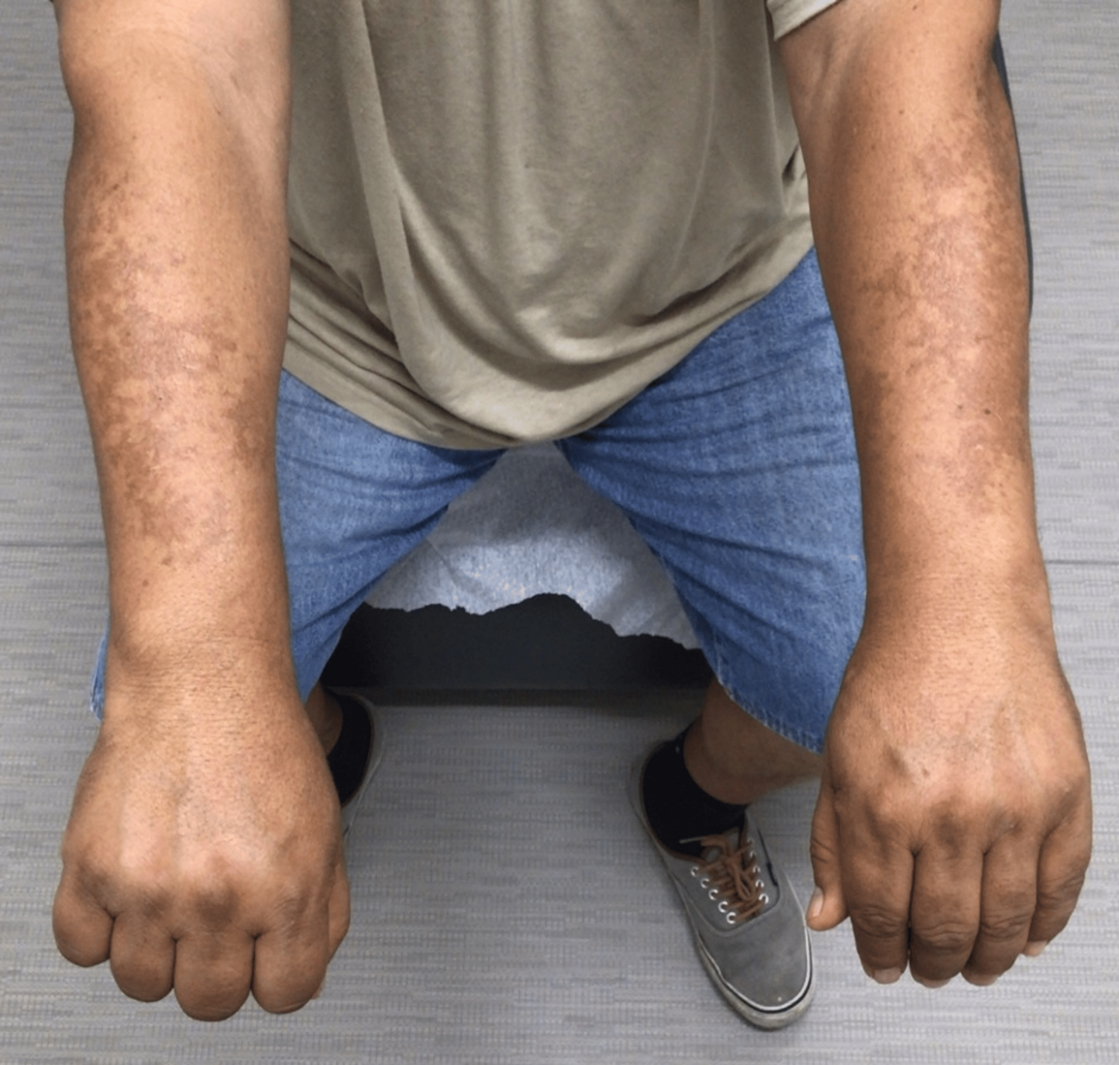
Symmetric hyperpigmented brown patches with irregular, moth-eaten borders involving the bilateral forearms, consistent with extra-facial melasma

A 66-year-old Hispanic male presented to the clinic for evaluation of asymptomatic hyperpigmentation involving his bilateral forearms, which had been progressively present for approximately one year. Physical examination revealed symmetric, large brown patches with irregular, moth-eaten borders distributed over both forearms (Figure 1). The patient worked as a truck driver and reported extensive chronic sun exposure to his upper extremities during long driving routes. Notably, there was no facial hyperpigmentation. However, he reported a history of a motor vehicle accident two years prior that resulted in second-degree burns involving the entire facial region.

**Figure 2:**
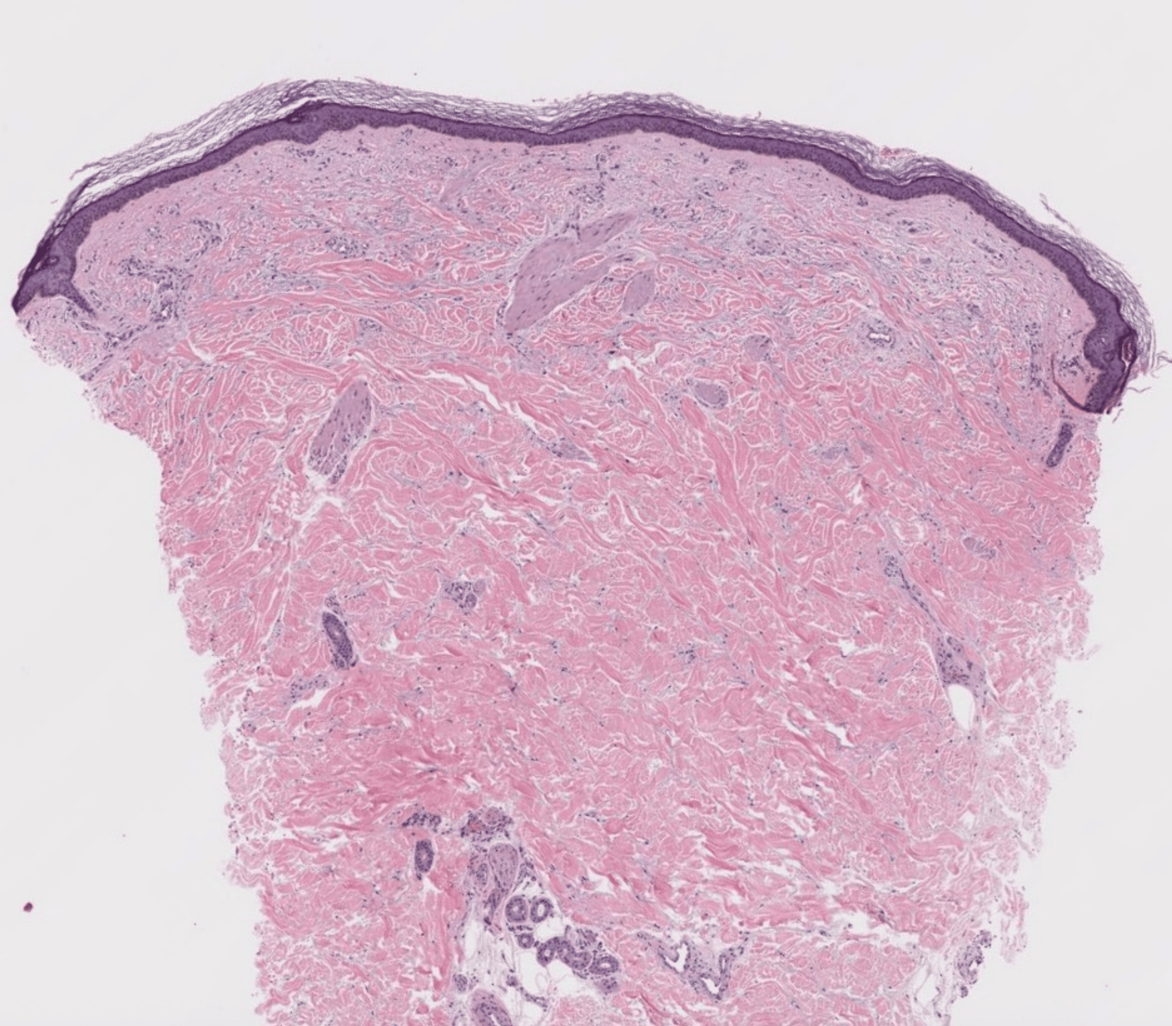
Hematoxylin and eosin (H&E)–stained section from a 4-mm punch biopsy of the right forearm. Low-power magnification (2x) demonstrating mild epidermal thinning with subtle architectural changes.

**Figure 3:**
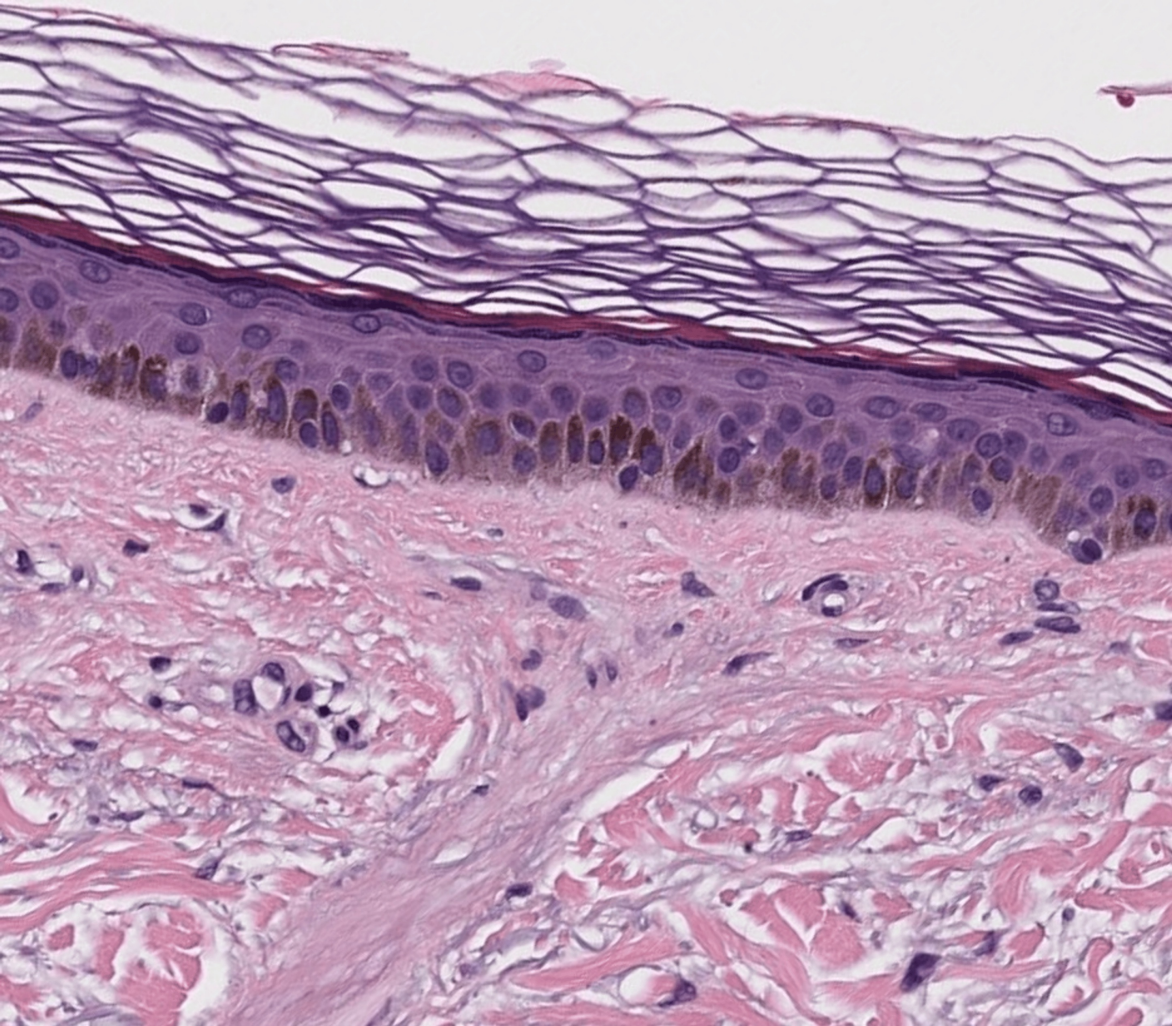
Hematoxylin and eosin (H&E)–stained section from a 4-mm punch biopsy of the right forearm. Higher-power magnification (20x) showing increased melanin pigmentation within basal keratinocytes and scattered dermal melanophages, consistent with epidermal melasma.

The differential diagnosis included melasma, erythema dyschromicum perstans, acquired brachial cutaneous dyschromatosis, exogenous ochronosis, confluent and reticulated papillomatosis, lichen planus pigmentosus, pigmented contact dermatitis, and post-inflammatory hyperpigmentation. A 4-mm punch biopsy was obtained from the right forearm. Histopathologic evaluation with hematoxylin and eosin staining demonstrated increased melanin pigment within basal keratinocytes of the epidermis (Figures 2, 3). Scattered melanophages were observed in the superficial dermis without associated inflammatory infiltrates. The epidermis showed mild thinning with effacement of rete ridges. These findings were most consistent with epidermal melasma.

The patient was counseled on strict photoprotection, including regular use of broad-spectrum sunscreen. Treatment with topical azelaic acid 15% daily was initiated. During subsequent telephone follow-up, the patient reported minimal improvement. Ongoing clinical follow-up was limited due to his occupational schedule and long-haul driving commitments.

## Discussion

Melasma is a common acquired pigmentary disorder that predominantly affects women and typically involves the face. In contrast, extra-facial melasma is less frequently reported and is most often described in postmenopausal women [2]. The presentation in this case - a male patient with isolated, symmetric involvement of the bilateral forearms - represents an uncommon clinical scenario and contributes to the limited literature describing melasma in men. Prior studies have shown that men account for a minority of melasma cases, with most demonstrating facial involvement [3,5]. Reports of exclusively extra-facial melasma in men remain exceedingly rare, though cases involving the forearms have been described [7].

Chronic UV radiation is a well-established driver of melanogenesis and plays a central role in the pathogenesis of melasma [4]. Occupational UV exposure has been increasingly recognized as a contributing factor, particularly in men, outdoor workers, and drivers. Studies evaluating these occupational exposures demonstrate increased photodamage and pigmentary disorders in chronically sun-exposed skin [6]. While unilateral dermatoheliosis is classically described in drivers, the symmetric distribution on forearms observed in this case may reflect cumulative bilateral exposure and reflected UV radiation during prolonged driving.

The absence of facial involvement in this patient is particularly notable, given that the face is the most commonly affected site in melasma. A unique aspect of this case is the patient’s history of second-degree facial burns prior to the onset of forearm pigmentation. Although there is limited literature directly addressing melasma development in previously burned skin, thermal injury is known to result in long-term alterations in melanocyte density, function, and dermal remodeling [8]. It is plausible that these changes reduced melanocytic responsiveness in the affected facial skin, thereby sparing this region from melasma. This observation raises the possibility that local cutaneous injury may influence susceptibility to pigmentary disorders.

Histopathologic evaluation in this case demonstrated increased melanin within basal keratinocytes with minimal dermal melanophages and no significant inflammation, consistent with epidermal melasma. These findings are in keeping with previously described histopathologic features [9]. The absence of lichenoid inflammation or significant pigment incontinence helps distinguish melasma from other entities in the differential diagnosis, such as lichen planus pigmentosus and erythema dyschromicum perstans. Similarly, the lack of exogenous ochre pigment deposition and clinical history argues against exogenous ochronosis.

Management of melasma remains challenging, particularly in patients with ongoing UV exposure. Standard therapies include strict photoprotection and topical depigmenting agents such as hydroquinone, retinoids, tranexamic acid, thiamidol, kojic acid, and azelaic acid [5,10]. Recurrence and treatment resistance are common and well-documented in the setting of continued UV exposure [1]. In this case, limited response likely reflected persistent occupational sun exposure and inconsistent follow-up.

## Conclusions

This case highlights an uncommon presentation of extra-facial epidermal melasma in a male patient with significant occupational UV exposure and sparing of previously burned facial skin. Compared with existing literature, this case reinforces the central role of UV radiation in melasma pathogenesis while suggesting that prior cutaneous injury may modify local susceptibility to melanogenesis. Recognition of atypical presentations is important for accurate diagnosis and emphasizes the need to integrate clinical history, environmental exposure, and histopathologic findings in evaluation and management.
